# Transformer-Based Deep Learning for Preoperative Prediction of Microvascular Invasion in Hepatocellular Carcinoma

**DOI:** 10.3390/cancers17203314

**Published:** 2025-10-14

**Authors:** Ruilin He, Huilin Chen, Wenjie Zou, Mengting Gu, Xingyu Zhao, Ningyang Jia, Wanmin Liu

**Affiliations:** 1 School of Health Science and Engineering, University of Shanghai for Science and Technology, Shanghai 200093, China; hrlhrl16604614243@163.com; 2 Department of Radiology, Eastern Hepatobiliary Surgery Hospital, The Third Affiliated Hospital of Shanghai Naval Military Medical University, Shanghai 200438, China; chlyouxiang123@163.com; 3 Department of Radiology, Tongji Hospital, School of Medicine, Tongji University, Shanghai 200092, China; zouwenjie2000712@163.com (W.Z.); gmt_wy@163.com (M.G.); zxy101186@163.com (X.Z.); 4 Department of Radiology, Ruijin Hospital, Shanghai Jiao Tong University School of Medicine, Shanghai 200025, China

**Keywords:** hepatocellular carcinoma, microvascular invasion, Transformer model, radiomics, deep learning, multimodal integration

## Abstract

**Background**: Microvascular invasion (MVI) is a critical prognostic factor in hepatocellular carcinoma (HCC), but preoperative three-class prediction remains challenging. Radiomics and clinical biomarkers may enable more accurate and individualized assessment. **Aim**: The aim of this study was to develop and validate a Transformer-based deep learning framework that integrates radiomic and clinical features for direct three-class MVI classification in HCC patients. **Methods**: This retrospective study included 437 patients with pathologically confirmed hepatocellular carcinoma (HCC) and microvascular invasion (MVI) status from two campuses of a single institution. Patients from Hospital A (*n* = 305) were randomly divided into training and internal test cohorts, while patients from Hospital B (*n* = 132) were used as an independent external validation cohort. Radiomic features were extracted from preoperative Gd-BOPTA-enhanced MRI, and clinical laboratory data were collected. A two-stage feature selection strategy, combining univariate statistical testing and recursive feature elimination, was applied. A Transformer-based model was built to classify three MVI categories (M0, M1, M2), and its performance was evaluated in both the internal test cohort and the external validation cohort. Results were compared with those from traditional machine learning models, including Random Forest, Logistic Regression, XGBoost, and LightGBM. **Results**: On the internal test set (*n* = 76, Hospital A), the model achieved an accuracy of 0.733 (95% CI: 0.64–0.83), a weighted F1-score of 0.733, and a macro-average AUC of 0.880 (95% CI: 0.807–0.953). The sensitivity and specificity for M1 were 0.56 (95% CI: 0.31–0.78) and 0.86 (95% CI: 0.74–0.94), respectively; for high-risk M2 cases, the sensitivity was 0.73 (95% CI: 0.64–0.81) and the specificity was 0.91 (95% CI: 0.85–0.96). On the external validation set (*n* = 132, Hospital B), performance remained stable with an accuracy of 0.758, a weighted F1-score of 0.768, and a macro-average AUC of 0.886 (95% CI: 0.833–0.940). **Conclusions**: This Transformer-based model enables accurate and objective three-class MVI prediction using multi-modal features, supporting individualized surgical planning and improved clinical outcomes. In particular, the ability to preoperatively identify high-risk M2 patients may inform surgical margin design, guide adjuvant therapy strategies, and influence liver transplantation eligibility.

## Simple Summary

Microvascular invasion (MVI) is a key predictor of recurrence in hepatocellular carcinoma (HCC). We developed a Transformer-based deep learning model to noninvasively predict three MVI grades (M0, M1, and M2) using preoperative MRI radiomics and clinical biomarkers. The model achieved high accuracy across independent datasets. Notably, reliable identification of high-risk M2 patients before surgery could help optimize resection margins, guide adjuvant therapy, and support individualized treatment planning.

## 1. Introduction

Hepatocellular carcinoma (HCC) is the fifth most common cancer globally and the third leading cause of cancer-related mortality [1,2]. In the Asia–Pacific region, particularly China, chronic hepatitis B virus (HBV) infection accounts for 70–90% of HCC cases [3]. Although surgical resection and liver transplantation offer potential curative options for early-stage HCC, recurrence rates remain high, affecting 40–70% of patients post-resection and 10–15% post-transplantation, with most recurrences occurring within two years [4,5].

Microvascular invasion (MVI), defined as the presence of tumor cells within microvessels lined by endothelial cells, is widely recognized as a major predictor of early recurrence and poor prognosis after surgery [6,7]. International guidelines such as those from the American Association for the Study of Liver Diseases (AASLD) and the European Association for the Study of the Liver (EASL) identify MVI as a critical prognostic factor [2], although detailed subclassification has not been standardized globally. According to histopathological consensus, particularly the Liver Cancer Pathology Group of China (LCPGC) proposal [8], MVI is commonly categorized into three subtypes: M0 (no invasion), M1 (≤5 invaded vessels within 1 cm from the tumor margin), and M2 (>5 invaded vessels or invasion beyond 1 cm). Previous studies have demonstrated that M2 status is strongly associated with increased recurrence risk and inferior long-term survival [6]. Therefore, accurate preoperative identification of high-risk M2 patients could inform surgical margin design, guide adjuvant therapies, and potentially influence eligibility for liver transplantation.

Currently, MVI diagnosis relies exclusively on postoperative pathological examination, limiting its utility for preoperative decision-making [9]. To address this limitation, radiomics- and MRI-based predictive models have emerged as promising noninvasive alternatives. However, most existing studies are restricted to binary classification (MVI presence vs. absence) and lack the capacity to differentiate high-risk M2 status [10,11,12]. Although these approaches often report high AUC values (~0.90), they fail to differentiate M1 from M2, thereby limiting their clinical applicability. Only a few studies have attempted direct three-class prediction. Nevertheless, these models remain underexplored and often rely on conventional machine learning algorithms with limited ability to capture complex feature dependencies.

Radiomics enables the extraction of high-throughput, quantitative features from medical images, offering insights into tumor heterogeneity that is imperceptible to the human eye [13]. When combined with clinical laboratory indicators, radiomics can offer a comprehensive characterization of tumor biology. Nevertheless, the high dimensionality, redundancy, and complex interactions among these features pose challenges for conventional machine learning approaches [14].

Transformer models, originally developed for natural language processing (NLP) tasks, have recently demonstrated superior performance in modeling high-dimensional structured data through their self-attention mechanisms [15,16]. Their ability to capture nonlinear and hierarchical feature dependencies makes them particularly suitable for integrating multi-modal data sources, such as radiomic features and clinical variables, which traditional models often struggle with. In addition to their established role in characterizing macroscopic tumor morphology, radiomic features have been shown to reflect microscopic pathological properties, such as angiogenesis, stromal composition, and local immune infiltration. Prior studies have demonstrated significant correlations between quantitative MRI-derived texture features and histopathological findings of microvascular invasion in HCC, supporting the biological plausibility of imaging-based prediction of microscopic phenomena. This cross-modality association underpins the rationale for leveraging radiomics and deep learning models to noninvasively approximate MVI status.

In this study, we propose a Transformer-based deep learning model that integrates radiomic features from preoperative MRI and clinical laboratory data for direct three-class MVI classification (M0, M1, M2) in HCC patients. Our approach has the potential to facilitate more detailed risk stratification in a relatively automated and reproducible manner.

## 2. Materials and Methods

### 2.1. Patient Enrollment and Clinical Data Collection

This study was approved by the Ethics Committee of Eastern Hepatobiliary Surgery Hospital (approval number: EHBHKY2022-H-P002; approval date: 26 October 2022). The requirement for informed consent was waived due to the retrospective nature of the study. Patient privacy was strictly protected through anonymization procedures.

A total of 437 patients with pathologically confirmed hepatocellular carcinoma (HCC) and available microvascular invasion (MVI) assessments were included between January 2017 and December 2023. Of these, 305 patients from Hospital A were used for model training and internal testing (229 for training and 76 for testing), while 132 patients from Hospital B served as an independent external validation cohort. According to standardized pathological criteria, the final cohort comprised 267 M0 cases, 103 M1 cases, and 67 M2 cases.

The inclusion criteria were as follows: patients who underwent liver resection and received Gd-BOPTA-enhanced MRI within two months prior to surgery; histopathological confirmation of HCC and MVI status; no prior treatments before surgery (e.g., radiofrequency ablation, transarterial chemoembolization (TACE)); absence of extrahepatic malignancies; availability of complete clinical, imaging, and pathological data.

Exclusion criteria included patients who underwent preoperative local therapies, had extrahepatic malignancies, or lacked essential imaging or clinical data.

**Figure 1:**
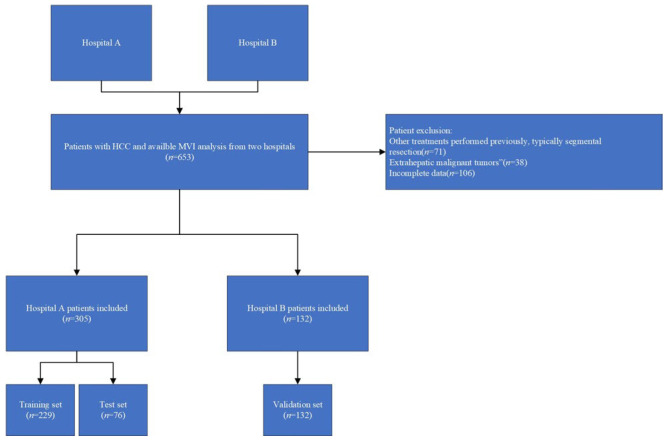
The workflow of patient selection. HCC: hepatocellular carcinoma; MVI: microvascular invasion.

Baseline demographic and clinical characteristics, including routine laboratory indicators (e.g., AFP, PIVKA-II, CA19-9, CEA, ALT, AST, ALB, TBIL, DBIL, GGT, PLT, PT), are summarized in Table 1. Liver function status was assessed based on the Child–Pugh classification, and all enrolled patients were classified as Child–Pugh class A or B before surgery. No statistically significant differences were observed among the training, internal test, and external validation cohorts. These clinical variables were selected based on their established relevance to tumor aggressiveness, liver function, and microvascular invasion, as supported by prior studies. They were used in conjunction with radiomic features as model inputs to provide complementary biological and imaging information. The patient selection process is illustrated in Figure 1.

### 2.2. MRI Image Acquisition

All participants were required to fast for at least 6 h and to abstain from water intake for at least 4 h before the MRI examination. The scanning range extended from the upper edge to the lower edge of the liver.

The contrast agent, 0.1 mmol/kg of Gd-BOPTA (MultiHance, Bracco Imaging S.p.A., Milan, Italy), was administered through injection into the median vein of the patient’s elbow using a high-pressure syringe at a rate of 2.0 mL/s, followed by a 20 mL saline flush lasting approximately 20 s.

After contrast agent administration, imaging was performed during specific phases: arterial phase (AP) at 25–45 s, portal venous phase (PVP) at 50–70 s, and delayed phase (DP) at 100–180 s. The MRI protocol also included fat-suppressed T2-weighted imaging (T2WI), T1-weighted imaging (T1WI), and diffusion-weighted imaging (DWI). To assess interobserver reproducibility, intraclass correlation coefficients (ICCs) were calculated for a subset of cases. Features with ICC > 0.75 were retained.

### 2.3. Imaging Analysis

Manual segmentation of HCC lesions was performed in a three-dimensional (3D) manner across all MRI sequences, including T2WI, T1WI, DWI, AP, PVP, and DP. Multiple contiguous slices covering the entire tumor volume were delineated to generate volumetric regions of interest (VOIs). Two radiologists (each with >5 years of experience in abdominal MRI interpretation) independently performed the segmentation, and a senior radiologist (>10 years of experience) reviewed the results. All radiologists were blinded to the pathological outcomes to avoid potential bias. Manual segmentation was conducted using ITK-SNAP software (version 3.6.0; http://www.itksnap.org, accessed on 1 October 2025). The consensus VOIs were subsequently used for radiomics feature extraction and statistical analysis. No qualitative radiological assessments were included; all model inputs consisted solely of objective data, including clinical laboratory parameters and quantitative radiomic features extracted from manually delineated VOIs. This strategy minimized potential interobserver variability and enhanced the objectivity, reproducibility, and generalizability of the predictive model.

### 2.4. Pathological Evaluation

All hematoxylin and eosin (H&E)-stained sections were retrospectively reviewed for MVI by two board-certified pathologists (each with >10 years of hepatopathology experience). Both pathologists were blinded to all clinical and imaging data, and discrepancies were resolved by consultation with a senior pathologist (>20 years of experience).

MVI status was determined according to the standardized histopathological criteria proposed by the Liver Cancer Pathology Group of China (LCPGC). Patients were classified into three subgroups: M0 (no microvascular invasion), M1 (≤5 invaded vessels within 1 cm of the tumor margin), and M2 (>5 invaded vessels or invasion beyond 1 cm).

This pathological classification (M0, M1, and M2) has been widely adopted and recognized in Chinese clinical practice. Although it has not yet been globally standardized, it demonstrates good consistency with histopathological assessments of microvascular invasion and is suitable for our study cohort based on Chinese patients.

Based on these criteria, the present cohort consisted of 267 M0 cases, 103 M1 cases, and 67 M2 cases. This pathology-based classification system has been widely adopted in clinical research for prognostic stratification of HCC.

### 2.5. Radiomic Feature Extraction

Radiomic feature extraction was conducted following manual segmentation of the regions of interest (VOIs) by an experienced radiologist using ITK-SNAP software (version 3.6.0) [17,18]. The delineated VOIs were used to extract quantitative features with the PyRadiomics (version 3.0.1, Computational Imaging Biomarker Group, Harvard Medical School, Boston, MA, USA) library. A total of 1500 radiomic features were obtained, including first-order statistical descriptors (such as mean, skewness, and kurtosis), shape-based metrics (such as sphericity and elongation), and texture features derived from gray-level matrices, including the Gray-Level Co-occurrence Matrix (GLCM) and the Gray-Level Run-Length Matrix (GLRLM) [18]. To better characterize tumor heterogeneity, features were extracted not only from the original images but also from filtered versions processed via wavelet and Laplacian of Gaussian (LoG) transformations [19,20]. These features collectively capture tumor intensity distributions, morphological characteristics, and intratumoral textural heterogeneity, which have been linked to histopathologic manifestations such as necrosis, angiogenesis, and stromal composition.

### 2.6. Data Preprocessing

Preprocessing involved two major steps:

- Missing Value Handling: Samples with more than 10% missing values were excluded. For the remaining samples, missing values were imputed separately for each feature using k-nearest neighbor (KNN) imputation (*k* = 3) implemented in Scikit-learn (version 1.3.1, Python Software Foundation, Wilmington, DE, USA)., based on the Euclidean distance in the feature space [21,22].
- Normalization: Radiomic features were normalized using Z-score transformation. The mean and standard deviation were calculated exclusively from the training set and then applied to the validation and test sets to ensure no information leakage during model evaluation [23]. Normalization parameters were derived from the training set and applied unchanged to the test and validation sets.

### 2.7. Feature Selection

To mitigate the risk of overfitting and enhance model generalizability, a two-stage feature selection strategy was employed. In the initial screening phase, features were first evaluated for reproducibility using the intraclass correlation coefficient (ICC) [19], and only those with ICC > 0.75 were retained. Subsequently, univariate statistical tests (*t*-test or Mann–Whitney U test, depending on the data distribution) were performed to exclude features with weak discriminative ability.

In the dimensionality reduction phase, recursive feature elimination (RFE) [24] based on Random Forest classifiers was conducted to further select the most informative subset of features.

**Figure 2:**
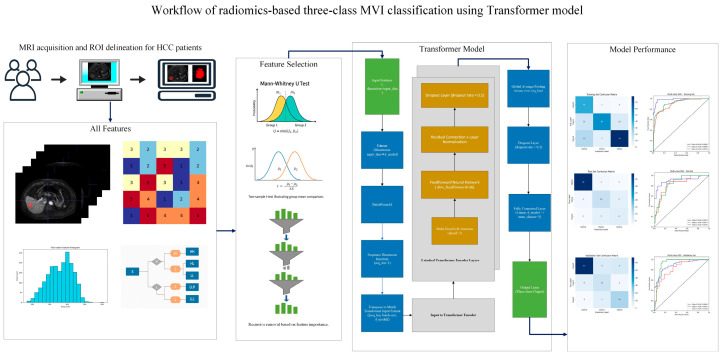
The overall workflow for radiomics-based three-class classification of microvascular invasion (MVI) in hepatocellular carcinoma (HCC). Radiomic features were extracted from Gd-enhanced MRI based on manually delineated regions of interest (VOIs). A two-step feature selection strategy was applied, combining univariate statistical tests (*t*-test or Mann–Whitney U test) and recursive feature elimination (RFE). A Transformer model was trained for MVI classification (M0, M1, M2), and performance was evaluated using confusion matrices and ROC curves.

To systematically evaluate the impact of different feature selection strategies, sixteen pipelines (4 initial screening methods × 4 dimensionality reduction methods) were constructed and compared using cross-validation performance within the training set. Ultimately, the pipeline combining “*t*-test or Mann–Whitney U test + RFE with Random Forest” was selected for final model training, as it achieved the best balance between feature compactness and model performance.The overall workflow of radiomics-based three-class MVI classification using the Transformer model is shown in Figure 2.

### 2.8. Model Architecture and Training

**Figure 3:**
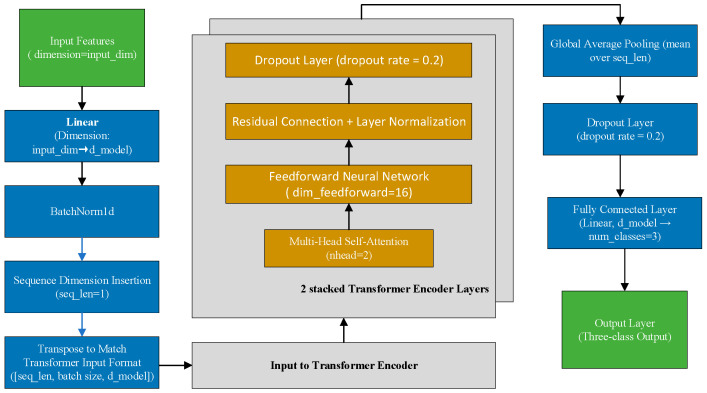
An overview of the Transformer-based model architecture for MVI classification.

We developed a Transformer-based deep learning model tailored for three-class MVI prediction. The model architecture was adapted from the Transformer framework, originally introduced for natural language processing tasks [15] and later extended to structured tabular data such as clinical and radiomic features [16]. The model was implemented in PyTorch (version 2.6.0, Meta AI, Menlo Park, CA, USA).As illustrated in Figure 3, the model architecture comprised three main components: an input embedding layer that projected the input features into an 8-dimensional latent space; two stacked Transformer encoder layers, each utilizing 2 attention heads, a feedforward dimension of 16, and a dropout rate of 0.2; and a final output classification layer that generated predictions across three categories.

Following the definition of the model architecture, we proceeded to model training. Cross-entropy loss with class-balanced weights was employed to mitigate class imbalance. Model parameters were optimized using the AdamW optimizer with a learning rate of 0.0005 and a weight decay of 0.02. The mini-batch size was set to 16. Training was conducted for 2000 epochs on an NVIDIA RTX 4090 GPU (NVIDIA Corporation, Santa Clara, CA, USA).

### 2.9. Data Augmentation and Balancing

**Figure 4:**
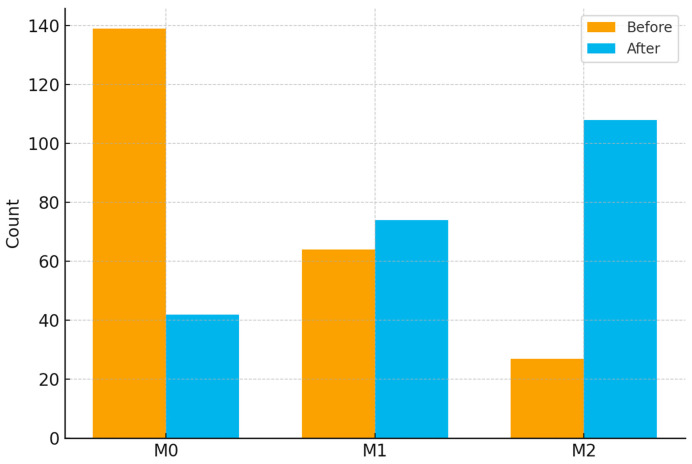
Effect of SMOTE-ENN on class distribution of MVI categories.

As illustrated in Figure 2 and Figure 3, the overall preprocessing and modeling pipeline included feature normalization, missing value imputation, two-step feature selection, and Transformer-based classification. To address the pronounced class imbalance in the training set, we employed a hybrid resampling strategy. Specifically, the Synthetic Minority Over-sampling Technique combined with Edited Nearest Neighbors (SMOTE-ENN) was applied to simultaneously oversample minority classes and remove borderline or noisy samples [22]. Importantly, resampling was performed exclusively on the training cohort, while the internal test and external validation cohorts were kept unchanged to preserve the integrity of model evaluation. After resampling, the training set exhibited a more balanced distribution across the three MVI categories (M0, M1, and M2), as demonstrated in Figure 4. Updated class distributions were then used to recompute class weights, which were incorporated into the cross-entropy loss function to further mitigate residual imbalance during training. This combined strategy has been widely reported to improve classifier robustness in highly imbalanced multi-class medical datasets.

## 3. Results

### 3.1. Baseline Characteristics

The baseline characteristics of the included patients are presented in Table 1. The absence of significant inter-cohort differences indicates that the three datasets were comparable for subsequent model development and validation.

### 3.2. Overall Model Performance

The Transformer-based model achieved consistently high performance across both internal and external cohorts (Table 2). In the internal test cohort, the model reached an accuracy of 0.733, a weighted F1-score of 0.733, and a macro-average AUC of 0.880 (95% CI: 0.807–0.953). In the external validation cohort, performance remained stable, with an accuracy of 0.758, a weighted F1-score of 0.768, and a macro-average AUC of 0.886 (95% CI: 0.833–0.940). These findings confirm the model’s robustness and generalizability across independent datasets.

### 3.3. Class-Wise Diagnostic Performance

Class-specific results are summarized in Table 3. The model achieved the highest accuracy for M0 cases, with sensitivity above 0.79 across the cohorts. For M1 cases, the sensitivity was moderate (0.56 in the test set and 0.62 in the validation set), but the specificity remained above 0.85. Importantly, for high-risk M2 cases, the sensitivity reached 0.74 in the external validation set, while the specificity remained above 0.89, highlighting the model’s potential clinical utility in identifying patients at greatest risk of recurrence.

### 3.4. Visualization of Classification Outcomes

**Figure 5:**
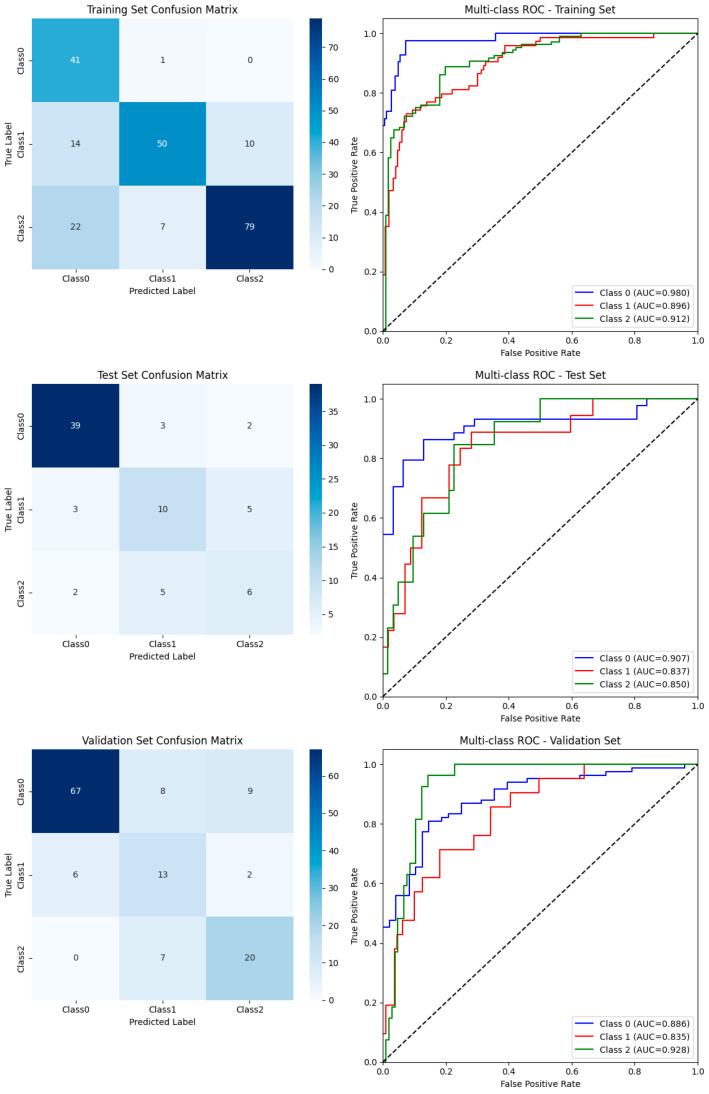
Classification performance of the Transformer-based model across the training, test, and validation sets. Top row: training set—confusion matrix (**left**) and ROC curves (**right**); middle row: test set—confusion matrix (**left**) and ROC curves (**right**); bottom row: external validation set—confusion matrix (**left**) and ROC curves (**right**). The diagonal dashed line in ROC plots represents random classification (*AUC* = 0.5).

The confusion matrices and ROC curves (Figure 5) illustrate classification outcomes across datasets. The model consistently showed strong discriminative ability for M0, whereas moderate overlap was observed between M1 and M2, reflecting the intrinsic difficulty of differentiating intermediate- and high-risk invasion.

### 3.5. Comparison with Traditional Machine Learning Models

Compared with traditional classifiers (Random Forest, Logistic Regression, XGBoost, and LightGBM), the Transformer-based model achieved superior performance across all metrics (Table 4). For example, it reached a validation AUC of 0.886 versus 0.834 for Random Forest and 0.837 for Logistic Regression. These results demonstrate the methodological advantage of the Transformer in handling high-dimensional, multi-modal data for MVI classification.

## 4. Discussion

Our adoption of a Transformer-based model in this context constitutes a methodological innovation in MVI classification. While Transformer architectures have been increasingly applied in medical imaging, their application to structured radiomic data for multi-class classification remains underrepresented. By tailoring the model to capture dependencies among quantitative features, our framework extends beyond prior binary MVI classification attempts, offering a more nuanced and clinically useful risk stratification strategy. Beyond methodological innovation, the proposed model also has practical clinical implications. Accurate preoperative identification of M2 cases may guide surgeons to consider wider resection margins, influence the decision to initiate adjuvant therapies, and potentially serve as an exclusion criterion for liver transplantation. Reliable recognition of M0 patients could prevent overtreatment and allow for more conservative surgical strategies. Importantly, distinguishing M1 cases provides additional value by identifying patients at intermediate risk, who may benefit from closer postoperative surveillance or tailored adjuvant treatment strategies. By providing this stratified information preoperatively, our framework has the potential to directly inform surgical planning and optimize patient management across all three subgroups.

This study demonstrated the feasibility and effectiveness of using a Transformer-based deep learning model to perform direct three-class classification of microvascular invasion (MVI) in hepatocellular carcinoma (HCC) patients by integrating radiomic features and clinical laboratory indicators. Compared with conventional machine learning methods such as Random Forest and Logistic Regression, the proposed model consistently achieved superior performance. Specifically, the Transformer obtained an AUC of 0.880 (95% CI: 0.807–0.953) and a weighted F1-score of 0.733 in the internal test cohort and maintained stable performance in the external validation cohort with an AUC of 0.886 (95% CI: 0.833–0.940) and a weighted F1-score of 0.768. These findings highlight not only the methodological advantages of the Transformer in modeling complex, high-dimensional, structured medical data but also its robustness and generalizability across independent datasets, attributable to its ability to capture nonlinear and hierarchical feature dependencies through its self-attention mechanism [15,16,25].

Previous radiomics-based studies have primarily focused on binary MVI classification (presence vs. absence), typically reporting AUCs ranging from 0.75 to 0.85 [11,26,27]. In contrast, our approach enables one-step, three-class prediction (M0, M1, M2), providing a more granular and clinically actionable preoperative risk stratification strategy. This advancement allows for refined surgical planning and supports individualized treatment decisions. Additionally, unlike earlier studies that often relied on subjective imaging features manually assessed by radiologists, our model is entirely based on objectively extracted features, which reduces interobserver variability and improves reproducibility [28].

The key contributions of this study lie in three aspects: proposing a Transformer-based framework for direct MVI three-class classification, demonstrating the benefit of multi-modal feature integration from radiomics and clinical data, and ensuring high model reproducibility by using only objective quantitative inputs. Nevertheless, several limitations must be acknowledged. First, although the model performed well on both internal and external validation sets, the dataset was collected from a limited geographic region, which may restrict its generalizability to broader populations and imaging protocols [29].

Future research should focus on external validation using multi-center and multi-ethnic datasets to improve robustness and generalizability [30]. In addition, incorporating multiomics data [e.g., genomics, transcriptomics, proteomics] and circulating tumor DNA (ctDNA) may further enhance the model’s predictive capacity and biological interpretability [31]. Recent evidence has shown that ctDNA can both predict and act independently of MVI [32], suggesting that its integration with imaging and clinical data represents a promising avenue for future research.

Nevertheless, several limitations should be acknowledged. First, this was a retrospective study conducted at two campuses of a single institution, which may limit generalizability to other populations and imaging protocols. Second, although the proposed Transformer model showed high performance, external validation using multi-center and multi-ethnic cohorts is required. Third, the radiomic features were extracted from manually segmented VOIs, which might have introduced observer bias despite quality control procedures. Future research should address these issues and explore integration with multiomics data to enhance model interpretability and clinical applicability.

The Transformer architecture inherently incorporates an attention mechanism, which offers potential for exploring feature importance and model interpretability. In future work, we plan to further investigate attention weight visualization to better understand the model’s decision process.

Although MVI serves as a crucial pathological biomarker associated with early recurrence and poor prognosis in HCC, it remains a surrogate rather than a direct clinical outcome.

Future work will focus on expanding the dataset through potential collaborations with other liver transplant centers, aiming to perform multi-center validation and improve the generalizability of the proposed Transformer-based model.

## 5. Conclusions

In this study, we proposed a Transformer-based deep learning framework that integrates radiomic features and clinical laboratory indicators for the preoperative three-class classification of MVI in patients with HCC.

The proposed model effectively captured complex high-dimensional feature relationships and achieved competitive performance across training, validation, and independent test sets.

By enabling direct three-class MVI prediction in a single step, our approach provides more granular prognostic information and holds promise for supporting individualized treatment planning and streamlining clinical decision-making.

Despite the encouraging results, further work is needed to enhance the model’s clinical applicability.

Future research should focus on expanding validation cohorts to larger, multi-center, and multi-ethnic datasets, improving model interpretability through explainable artificial intelligence (XAI) techniques, and incorporating multiomics data to enrich biological insights and further boost predictive accuracy.

## Figures and Tables

**Table 1:** Baseline demographic and clinical characteristics.

Characteristic	M0	M1	M2	*p*-Value
Demographics				
Age (years)	56.49 ± 10.43	55.21 ± 10.95	55.51 ± 9.56	0.52
Sex	227 (85.0%)	85 (82.5%)	55 (82.1%)	0.758
Tumor characteristics				
Tumor number	1.00 [1.00–1.00]	1.00 [1.00–1.00]	1.00 [1.00–1.00]	0.822
BCLC stage	156 (58.4%)	63 (61.2%)	35 (52.2%)	0.145
Child–Pugh stage	247 (93.2%)	100 (97.1%)	60 (89.6%)	0.122
AFP (ng/mL)	12.79 [3.54–163.90]	40.70 [9.54–544.92]	198.80 [28.70–1210.00]	<0.001
PIVKA-II (mAU/mL)	100.00 [31.00–553.25]	163.00 [35.75–839.25]	234.00 [81.00–2204.00]	<0.001
Laboratory indicators				
CA19-9 (U/mL)	16.40 [8.65–30.06]	14.20 [9.20–24.80]	12.45 [8.60–26.74]	0.323
CEA (ng/mL)	2.40 [1.60–3.42]	2.80 [1.50–4.07]	2.70 [1.80–3.60]	0.346
ALT (U/L)	28.00 [19.00–42.00]	25.50 [17.00–39.75]	30.00 [23.00–42.50]	0.316
AST (U/L)	26.00 [20.00–38.00]	26.00 [20.00–37.00]	26.00 [21.25–41.75]	0.408
Albumin (g/L)	42.00 [39.38–44.80]	41.55 [39.30–44.50]	42.35 [39.32–44.33]	0.995
TBIL (µmol/L)	13.75 [10.85–19.55]	14.60 [11.00–18.70]	14.50 [10.93–20.27]	0.918
DBIL (µmol/L)	5.35 [4.10–7.62]	5.70 [4.20–6.80]	5.80 [3.95–7.97]	0.839
IBIL (µmol/L)	8.10 [6.38–11.55]	9.15 [6.70–11.57]	8.40 [6.83–11.40]	0.582
GGT (U/L)	40.00 [25.75–83.00]	43.00 [26.00–79.75]	51.00 [28.50–111.25]	0.456
Cholinesterase (U/L)	6906.82 ± 1783.92	6997.93 ± 1737.74	6885.03 ± 1826.85	0.903
Total protein (g/L)	68.65 ± 5.75	68.64 ± 4.95	67.03 ± 5.13	0.088
Cholesterol (mmol/L)	3.75 [3.27–4.38]	3.78 [3.38–4.28]	4.11 [3.57–4.59]	0.187
Triglyceride (mmol/L)	1.01 [0.78–1.31]	1.02 [0.80–1.38]	1.04 [0.88–1.33]	0.596
HDL-C (mmol/L)	1.15 [0.97–1.38]	1.05 [0.91–1.32]	1.04 [0.84–1.23]	0.015
LDL-C (mmol/L)	2.33 [1.93–2.84]	2.44 [2.07–2.71]	2.63 [2.20–3.18]	0.02
HBsAg	220 (82.4%)	94 (91.3%)	56 (83.6%)	0.102
HBsAb	40 (15.0%)	13 (12.6%)	12 (17.9%)	0.637
HBeAg	48 (18.0%)	23 (22.3%)	13 (19.4%)	0.635
HBeAb	185 (69.3%)	67 (65.0%)	48 (71.6%)	0.622
HBcAb	253 (94.8%)	101 (98.1%)	64 (95.5%)	0.32
HBV DNA	260 (97.4%)	100 (97.1%)	65 (97.0%)	0.98

**Table 2:** Overall performance of the proposed Transformer-based model.

Dataset	Accuracy	Weighted F1-Score	Macro-Average Precision	Macro-Average Recall	Macro-Average AUC (95% CI)
Training Set	0.759	0.766	0.761	0.794	0.920 [0.884–0.955]
Test Set	0.733	0.733	0.635	0.635	0.880 [0.807–0.953]
Validation Set	0.758	0.769	0.676	0.719	0.886 [0.833–0.940]

**Table 3:** Class-wise diagnostic performance.

Dataset	Class	Support	Sensitivity (95% CI)	Specificity (95% CI)
Training	M0	42	0.976 [0.87–1.00]	0.802 [0.74–0.86]
Training	M1	74	0.676 [0.56–0.78]	0.9467 [0.90–0.98]
Training	M2	108	0.731 [0.64–0.81]	0.914 [0.85–0.96]
Test	M0	44	0.886 [0.75–0.96]	0.839 [0.66–0.95]
Test	M1	18	0.556 [0.31–0.78]	0.860 [0.74–0.94]
Test	M2	13	0.462 [0.19–0.75]	0.887 [0.78–0.95]
Validation	M0	84	0.798 [0.70–0.88]	0.875 [0.75–0.95]
Validation	M1	21	0.619 [0.38–0.82]	0.865 [0.79–0.92]
Validation	M2	27	0.895 [0.54–0.95]	0.8952 [0.82–0.95]

**Table 4:** Comparison of the proposed Transformer-based model with traditional machine learning models.

Model	Validation Accuracy	Validation F1-Score	Validation AUC	Test Accuracy	Test F1-Score	Test AUC
Random Forest	0.682	0.694	0.834	0.640	0.660	0.827
Logistic Regression	0.629	0.638	0.837	0.547	0.575	0.802
XGBoost	0.545	0.568	0.807	0.533	0.557	0.804
LightGBM	0.523	0.547	0.800	0.573	0.596	0.823
Transformer (ours)	0.758	0.769	0.886	0.733	0.733	0.880

## Data Availability

The datasets generated and/or analyzed during the current study are not publicly available due to patient privacy restrictions but are available from the corresponding author upon reasonable request.

## Abbreviations

HCC	Hepatocellular carcinoma
MVI	Microvascular invasion
MRI	Magnetic resonance imaging
AUC	Area under the curve
Gd-BOPTA	Gadolinium benzyloxypropionictetraacetate
RFE	Recursive feature elimination
